# Cytoreductive Surgery Plus HIPEC in Recurrent or Newly Diagnosed Advanced Epithelial Ovarian Cancer: a Meta-analysis

**DOI:** 10.1245/s10434-025-16979-6

**Published:** 2025-02-04

**Authors:** C. Taliento, S. Restaino, M. Arcieri, G. Scutiero, P. Greco, G. Scambia, G. Vizzielli

**Affiliations:** 1 Department of Medical Sciences, Institute of Obstetrics and Gynecology, University of Ferrara, Ferrara, Italy; 2 Clinic of Obstetrics and Gynecology, “Santa Maria della Misericordia” University Hospital, Azienda Sanitaria Universitaria Friuli Centrale, Udine, Italy; 3 PhD School in Biomedical Sciences, Gender Medicine, Child and Women Health, University of Sassari, Sassari, Italy; 4 Dipartimento per la salute della Donna e del Bambino e della Salute Pubblica, UOC Ginecologia Oncologica, Fondazione Policlinico Universitario A. Gemelli IRCCS, Rome, Italy; 5 Università Cattolica del Sacro Cuore, Rome, Italy; 6 Department of Medicine (DMED), University of Udine, Udine, Italy

**Keywords:** HIPEC, Ovarian cancer, Cytoreductive surgery, Adverse events, Progression-free survival, PFS

## Abstract

**Background:**

In 2024, two randomized controlled trials (RCTs) were published, providing new high-quality evidence on HIPEC in epithelial ovarian cancer (EOC). Updating data on progression-free survival (PFS) and adverse events could offer a clearer understanding of the benefits and risks of HIPEC combined with cytoreductive surgery (CRS), with or without prior neoadjuvant chemotherapy (NACT).

**Patients and Methods:**

An electronic search was conducted using PubMed, Web of Science, EBSCO, and CENTRAL up to 23 November 2024. We only included RCTs reporting PFS and adverse events of interval or secondary CRS, with or without HIPEC, in newly diagnosed or recurrent EOC.

**Results:**

The meta-analysis included six RCTs. The addition of HIPEC to surgery significantly improved PFS in patients with newly diagnosed advanced-stage EOC who received NACT (HR 0.59; 95% CI 0.39–0.88; *p* = 0.01). No significant difference in PFS was observed between secondary CRS plus HIPEC and CRS alone in recurrent ovarian cancer without prior NACT (HR 1.22; 95% CI 0.82–1.83; *p* = 0.32). Regarding adverse events, a decrease in platelet count of any grade was more frequent in the HIPEC group (*p* = 0.03). The overall risk of acute kidney failure (AKF) was 10.6%, with a significantly higher incidence compared with CRS alone (*p* = 0.003).

**Conclusions:**

The addition of HIPEC to CRS significantly improved PFS compared with surgery alone in patients with advanced EOC who received NACT. However, the treatment was associated with a higher incidence of AKF, which occurred in 10.6% of patients who underwent HIPEC.

**Supplementary Information:**

The online version contains supplementary material available at 10.1245/s10434-025-16979-6.

Ovarian cancer remains one of the leading causes of mortality among gynecological cancers, with its incidence steadily increasing, as indicated by Globocan’s 2020 projections.^1,2^ Over the past few decades, cytoreductive surgery (CRS) combined with hyperthermic intraperitoneal chemotherapy (HIPEC) has been proposed as a potential strategy to improve patient oncological outcomes.^3,4^ The therapeutic rationale of HIPEC relies on two mechanisms: the direct cytotoxic effect of heat on cancer cells and the enhancement of chemotherapy efficacy through thermal potentiation, which increases drug uptake.^5^ In addition, intraperitoneal administration of the drug can deliver high concentrations directly to the peritoneum, while limiting its toxic effects on other tissues, such as bone marrow.^6^

In 2018, one randomized controlled trial demonstrated that, among patients with stage III epithelial ovarian cancer, the addition of HIPEC to interval CRS resulted in longer recurrence-free survival and overall survival compared with surgery alone, without an increase in adverse events.^7^ However, the results of this trial have been widely debated, and to date, no consensus has been reached regarding the role of HIPEC in combination with CRS.^8^

Moreover, data on adverse events of HIPEC can be controversial in literature.^9,10^ For example, the overall frequency of renal failure after HIPEC in ovarian cancer can range from 8% in some studies to approximately 50% in another study.^11–13^ This is partially because most of the literature on HIPEC for the treatment of ovarian cancer is derived from retrospective studies, which are often associated with a high risk of bias.^11^ In 2024, two randomized controlled trials (RCTs), evaluating the efficacy and safety of HIPEC in combination with CRS, were published, providing new high-quality data on both the oncological outcomes and complications associated with HIPEC and surgery.^7,14–18^

Therefore, an updated systematic assessment of oncological outcomes, and chemotherapy and surgery-related complications is essential to better define the risk–benefit ratio of this approach.

## Patients and Methods

### Search Strategy, Outcomes, and Inclusion Criteria

A systematic search was performed, including the PubMed, Web of Science, EBSCO-host, and Cochrane Library databases up to 23 November 2024. The Preferred Reporting Items for Systematic Reviews and Meta-Analyses (PRISMA) checklist was followed to conduct this systematic review.^19^ The protocol was registered in PROSPERO (CRD42024618352). The full search strings for all databases can be found in Supplementary Material 1.

This meta-analysis of randomized controlled trials aims to evaluate progression-free survival (PFS) and the incidence of complications associated with HIPEC and CRS (interval or upfront) in comparison with CRS alone in patients with recurrent or newly diagnosed epithelial advanced ovarian cancer.

RCTs reporting PFS and adverse events of CRS (primary or interval debulking) with or without HIPEC in newly diagnosed or recurrent epithelial ovarian cancer were included. Two reviewers (C.T. and G.V.) independently reviewed the literature and screened the articles according to the predefined search strategy. Rayyan online software was used to eliminate duplicate records and screen titles and abstracts; following this, full-text articles of selected studies were retrieved. Any discrepancies in decisions regarding study inclusion were discussed by the authors until an agreement was reached.

### Data Extraction and Study Risk of Bias

The data were extracted into a standardized Excel form and included the following information: recruitment period, sample size, disease status, International Federation of Gynecology and Obstetrics (FIGO) stage, HIPEC regimens, PFS, and chemotherapy and surgery-related adverse events. Data extraction was conducted independently by two reviewers using a standardized data collection form. We focused on the toxicity of HIPEC using the National Cancer Institute Common Terminology Criteria for Adverse Events, version 4 (NCI-CTCAE v4). Any discrepancies were resolved through discussion with a third reviewer. Two reviewers (C.T. and G.V.) independently assessed the risk of bias in each study using the Cochrane Risk of Bias Assessment tool for randomized studies (RoB 2).

### Statistical Analysis

Statistical meta-analysis was performed using Review Manager software (RevMan, version 5.3, The Nordic Cochrane Centre, Copenhagen, Denmark, 2011). 95% confidence intervals (CI) for all calculated rates were given. Odds ratios (OR) were used in the analysis. PFS estimates were derived on the basis of the hazard ratios (HRs) provided. If patients treated with HIPEC were considered the reference group (HR = 1) in the original study, the reported HRs for other groups were inverted to align with the analysis framework, allowing for consistent interpretation of treatment effects. Heterogeneity of combined trials was determined by chi-squared and *I*^2^ tests. However, to account for heterogeneity between studies, a random-effects model was applied to all studies as a conservative measure.

## Results

### Study Selection

**Fig. 1:**
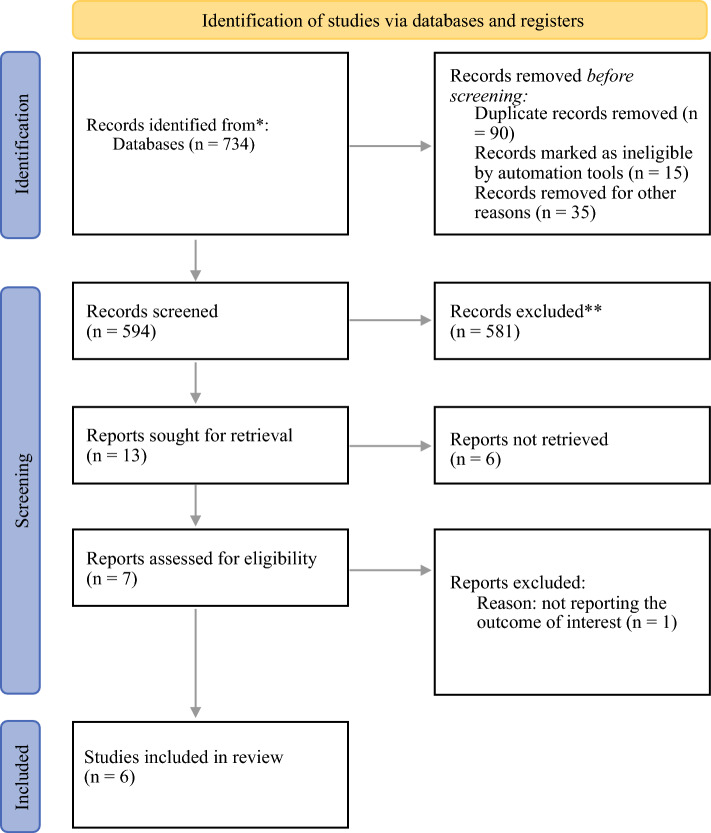
PRISMA flow chart

After screening abstracts and full texts, a total of six RCTs, published in peer-reviewed journals between 2018 and 2024, were included in the present meta-analysis.^7,14–18^ The study by Spiliotis et al. was excluded because complications were not reported.^20^ In the HIPEC cohort, there were 587 patients, while the non-HIPEC cohort included 628 patients. The PRISMA flow diagram shows the review process (Fig. 1).

### Study Characteristics

A total of three RCTs evaluated HIPEC in the relapse setting, while three RCTs evaluated HIPEC in patients with primary advanced EOC. Performance status criteria varied across the included studies, with three studies utilizing an Eastern Cooperative Oncology Group (ECOG) performance status of 0 or 1,^15–17^ one study applying a Karnofsky performance score of > 70%,^18^ and two studies employing a World Health Organization (WHO) performance status of < 2.^7,14^ Cisplatin, at doses ranging from 75 mg/m^2^, to 100 mg/m^2^, was the chemotherapeutic agent used in five of the six studies, while carboplatin was used exclusively by Zivanovic et al. at a dose of 800 mg/m^2^ (Table 2).^18^ The perfusion period was 60 min in three studies and 90 min in the remaining studies.

In three studies, a closed technique was used for HIPEC, while an open technique was performed in two RCTs. In the CHIPOR trial, 74% of HIPEC procedures were performed using the closed technique, and 25% using the open technique.^14^ To reduce the risk of cisplatin-related renal toxicity, sodium thiosulfate was administered in three studies.^7,14,16^

In all studies, except for Zivanovic et al., adverse events were reported using the National Cancer Institute Common Terminology Criteria for Adverse Events. Therefore, five RCTs provided data on grade ≥ 3 adverse events.

The monitoring of complications was conducted at different timepoints across the studies: in two studies, it was assessed at 30 days; in one study, at 60 days; and in the remaining three studies, from random assignment at the time of the surgery to 6 weeks after the completion of the last cycle of chemotherapy following HIPEC and surgery. The characteristics of the six trials are described in Table 1.

**Table 1:** Characteristics of the included randomized controlled trials

Study	Country, study period	Sample size HIPEC non-HIPEC		Randomization	Performance status, eligibility criteria	Disease status	Treatment	Grading system for adverse events	Complication monitoring	Technique	Nephroprotection with sodium thiosulfate
Van Driel 2018 ^7^	The Netherlands	122	123	At the time of surgery in cases with residual tumor ≤ 1 cm	WHO performance status < 2	Primary advanced (stage III)	IDS ± HIPEC and three cycles of carboplatin and paclitaxel	CTCAE v 4.0	From random assignment to 6 weeks after completion of last cycle of chemotherapy	100 mg/m^2^ cisplatin, 40 °C, 90 min, open	Administered at the start of perfusion as an intravenous bolus (9 g/m^2^ in 200 ml)
Zivanovic 2021 ^18^	USA	49	49	At the time of surgery in cases with residual tumor ≤ 0.5 cm	Karnofsky performance score of > 70%	Recurrence, platinum sensitive	SCS ± HIPEC and five or six cycles of carboplatin-based chemotherapy	MSKCC SSE grading system	Within 30 days after surgery	800 mg/m^2^ carboplatin, 41–43 °C, 90 min, closed	Not used
Cascales Campos 2021 ^17^	Spain	35	36	At the time of surgery in cases with residual tumor ≤ 0.25 cm	ECOG performance status of 0 or 1	Primary advanced	IDS ± HIPEC	CTCAE v 3.0	Within 30 days after surgery	75 mg/m^2^ cisplatin, 42–43 °C, 60 min, open	Not used
Lim 2022 ^16^	South Korea 2010–2016	92	92	At the time of surgery in cases with residual tumor ≤ 1 cm	ECOG performance status of 0 or 1	Primary advanced (stage III–IV)		CTCAE v 4.0	From random assignment to 6 weeks after completion of last cycle of chemotherapy	75 mg/m^2^ cisplatin, 41.5 °C, 90 min, closed	After protocol amendment in 21 patients
Fagotti 2024 (HORSE-MITO-18) ^15^	Italy	82	85	At the time of the surgery in cases with residual tumor ≤ 0.25 cm	ECOG performance status of 0 or 1	Recurrence, platinum sensitive, stage I–IV	SCS ± HIPEC	CTCAE v 4.0, MSKCC SSE grading system	From random assignment to 6 weeks after completion of last cycle of chemotherapy	75 mg/m^2^ cisplatin, 41.5 °C, 60 min, closed	Not used
Classe 2024 (CHIPOR) ^14^	France 2011–2021	207	208	At the time of surgery in cases with residual tumor ≤ 0.25 cm	WHO performance status < 2	Recurrence, stage III	IDS ± HIPEC and three cycles of carboplatin and paclitaxel	CTCAE v 4.0	Within 60 days after surgery	75 mg/m^2^ cisplatin, 41 ± 1 °C, 60 min, open	After protocol amendment in 2018

SCS, Secondary debulking surgery; NACT, neoadjuvant chemotherapy; PRS, post-recurrence survival; SSE, surgical secondary events; CTCAE, common terminology criteria for adverse events; ECOG, Eastern Cooperative Oncology Group; WHO, World Health Organization

### Progression-Free Survival

**Fig. 2:**
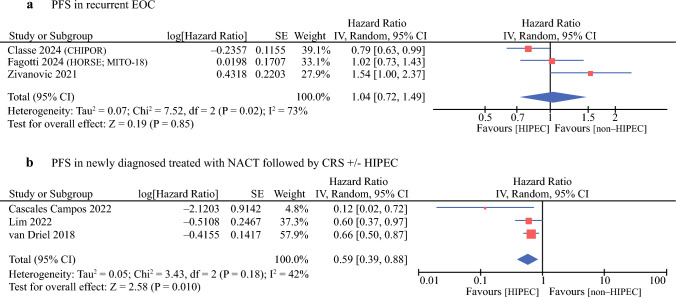
Forest plots of comparisons of 26 PFS outcomes: **a** PFS in recurrent epithelial ovarian cancer; **b** PFS in newly diagnosed advanced epithelial ovarian cancer treated with NACT followed by CRS ± HIPEC

We conducted subgroup meta-analyses on the basis of disease status. Three studies included patients with recurrent EOC. In the CHIPOR trial, patients who had been pretreated with six cycles of chemotherapy, with or without bevacizumab, were randomly assigned to receive HIPEC or not.^14^ Conversely, in the remaining two studies, none of the patients received chemotherapy before secondary CRS.^15,18^ Pooled analysis of these three trials showed no significant difference between the CRS plus HIPEC and CRS alone groups (HR 1.04; 95% CI 0.72–1.49; *p* = 0.85), with high heterogeneity (*I*^2 ^= 73%) (Fig. 2, Table 2). After excluding the study by Classe et al., the results of the subgroup analysis, which included only studies that did not use NACT, remained statistically insignificant (HR 1.22; 95% CI 0.82 – 1.83; *p* = 0.32).

**Table 2:** Synthesis of the results

Outcome	Number of RCTs	Total patients HIPEC non-HIPEC		Summary measure	95% CI	*p*-Value	*I*^2^%	Comment
PFS in primary setting	3	249	251	0.59	0.39–0.88	0.01	0	Favorable effect of HIPEC plus CRS
PFS survival in recurrent setting	3	338	342	1.04	0.72–1.49	0.85	73	No significant difference
Anemia	6	580	579	1.56	0.89–2.73	0.12	36	No significant difference
Platelet count decrease	4	427	421	1.55	1.03–2.33	0.03	12	Slightly higher risk with HIPEC
Neutrophil count decrease	2	171	164	1.02	0.40–2.61	0.97	74	No significant difference
White blood cell decrease	2	171	164	1.07	0.62–1.83	0.82	6	No significant difference
Fatigue	2	197	194	1.49	0.93–2.39	0.09	0	No significant difference
Nausea	4	338	335	1.26	0.88–1.80	0.21	0	No significant difference
Diarrhea	4	338	335	1.29	0.82–2.05	0.27	0	No significant difference
Vomiting	2	325	330	0.67	0.39–1.16	0.15	6	No significant difference
Constipation	3	289	286	0.73	0.48–1.11	0.14	0	No significant difference
Liver dysfunction	3	378	372	0.64	0.29–1.40	0.27	60	No significant difference
Acute kidney failure	4	416	421	4.01	1.62–9.96	0.003	26	Higher risk with HIPEC
Infections	6	583	592	1.06	0.74–1.52	0.74	4	No significant difference
Sepsis/septic shock	5	548	556	1.37	0.54–3.49	0.50	0	No significant difference
Hemorrhage	5	501	507	1.56	0.62–3.87	0.34	0	No significant difference
Lymphocele	4	416	421	0.59	0.27–1.30	0.19	0	No significant difference
Ileus/bowel (sub)occlusion	6	583	592	0.93	0.58–1.50	0.77	5	No significant difference

The meta-analysis, which included three studies enrolling patients with primary advanced EOC who had undergone NACT, revealed that CRS plus HIPEC affects PFS compared with CRS alone, with moderate heterogeneity across the studies (HR 0.59; 95% CI 0.39–0.88; *I*^2 ^= 42%). In the study by Lim et al., the HR included in the meta-analysis was derived from the subgroup of patients who received NACT, comprising 34 out of 92 patients in the HIPEC group and 43 out of 92 in the control group.

### Adverse Events

**Fig. 3:**
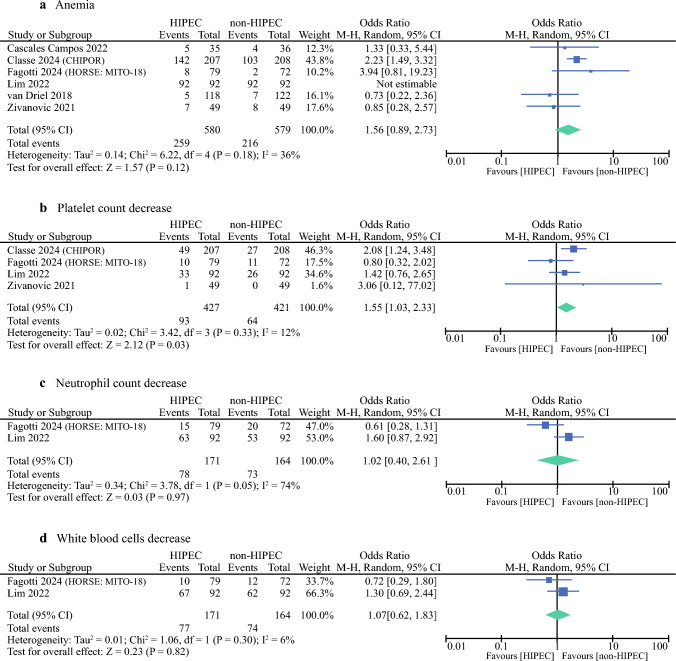
Hematological disorders (any grade) forest plots of comparisons: **a** anemia, **b** platelet count decrease, **c** neutrophil count decrease, and **d** white blood cell decrease

A total of 16 adverse events were available for statistical analysis in the six included RCTs. Common hematological disorders during follow up included anemia, platelet count decrease, white blood cell decrease, and neutrophil count decrease. Our statistical analysis results showed that compared with CRS alone, CRS followed by HIPEC had a similar rate of anemia of any CTCAE grade (OR 1.56; 95% CI 0.89–2.73; *p* = 0.012; *I*^2^ = 36%) (Fig. 3). Sensitivity analysis illustrated that the study by Classe et al. displayed an apparent influence on the overall result of the meta-analysis. After the exclusion of this study, the pooled OR was similar (OR 1.21; 95% CI 0.65–2.23; *p* = 0.55), but the heterogeneity was low (*I*^2^ = 8%). In addition, considering only anemia grade > 3 events, although the frequency was higher in HIPEC group, no statistically significant differences were observed (OR 1.43; 95% CI 1.00–2.04; *p* = 0.05).

Moreover, no statistically significant differences were observed for white blood cell decrease (OR 1.07; 95% CI 0.62–1.83; *p* = 0.82) or neutrophil count decrease (OR 1.02; 95% CI 0.40–2.61; *p* = 0.97). Similarly, the percentage of patients who had grade ≥ 3 of these blood disorders was similar between the two groups, as shown in Supplementary Material 2.

The pooled result from the random effects model showed that the rate of platelet count decrease (OR 1.55; 95% CI 1.03–2.33; *p* = 0.03; *I*^2^ = 12%) was higher in HIPEC group compared with non-HIPEC group. However, no differences were observed in terms of grade 3 or worse platelet count decrease (*p* = 0.88).

**Fig. 4:**
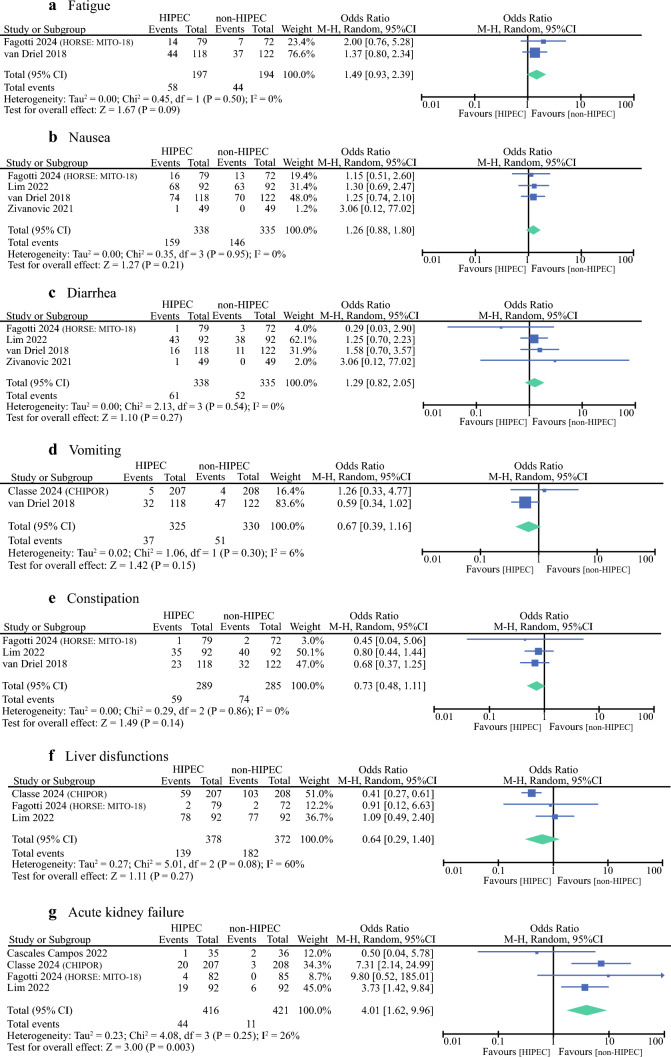
Other chemotherapy-related adverse events forest plots of comparisons: **a** fatigue, **b** nausea, **c** diarrhea, **d** vomiting, **e** constipation, **f** liver dysfunction, and **g** acute kidney failure

In total, four RCTs assessed diarrhea and nausea: ^7,15,16,18^ three of the four studies evaluated these complications up to 6 weeks after the completion of the last cycle of chemotherapy following CRS plus HIPEC or CRS alone ^7,15,16^ and one study assessed these complications within 30 days after surgery.^18^ No statistically significant differences were found in any of the studies, nor with meta-analysis results, for nausea (OR 1.26; 95% CI 0.88–1.80; *p* = 0.21; *I*^2^ = 0%) or diarrhea (OR 1.29; 95% CI 0.82–2.05; *p* = 0.27; *I*^2^ = 0%), pointing to similar adverse events rates in the two groups. Moreover, no differences were observed for fatigue (OR 1.49; 95% CI 0.93–2.39; *p* = 0.09; *I*^2^ = 0%), vomiting (OR 0.67, 95% CI 0.39–1.16; *p* = 0.15; *I*^2^ = 6%), or constipation (OR 0.73; 95% CI 0.48–1.11; *p* = 0.14; *I*^2^ = 0%), for either any grade or grade 3 or worse (Fig. 4; Table 2; Supplementary Material 3).

Liver dysfunction was recorded in three studies; two of them reported elevation of the aminotransferase enzymes, while one referred to hepatotoxicity, including cholestasis, gamma-glutamyl transferase increase, aspartate aminotransferase increase, transaminase increase, alanine aminotransferase increase, liver function test increase, drug-induced liver injury, and hepatic cytolysis.^14,16^ Pooling the data, the meta-analysis showed no significant differences between CRS versus CRS plus HIPEC (OR 0.64; 95% CI 0.29–1.40; *p* = 0.27; *I*^2^ = 60%).

Data on nephrotoxicity were reported by four trials.^14–17^ The overall incidence of acute kidney failure (AKF) was 10.6% (44/416). The meta-analysis, which included a total of 892 patients, demonstrated that CRS plus HIPEC was associated with a higher incidence of AKF compared with CRS alone (OR 4.01; 95% CI 1.62–9.96; *p* = 0.003; *I*^2^ = 26%) (Fig. 4, Table 2). Separate data on creatinine increase were provided by Lim et al., showing a creatinine increase of 68.5% in the HIPEC group compared with 47.8% in the control group.^16^

### Surgical Secondary Events

**Fig. 5:**
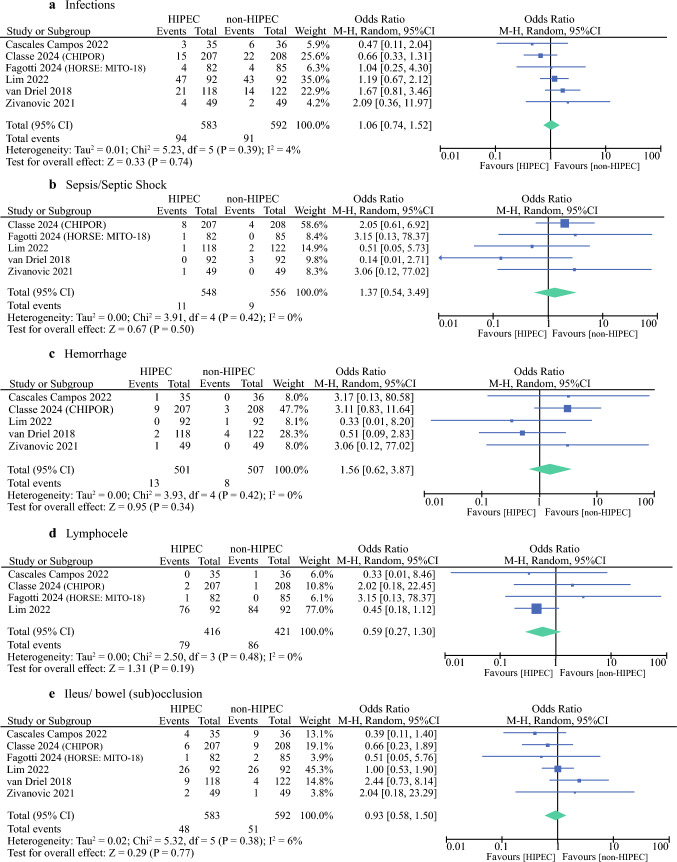
Surgical secondary events (any grade) forest plots of comparisons: **a** infections, **b** sepsis/septic shock, **c** hemorrhage, **d** lymphocele, and **e** ileus/bowel (sub) occlusion

Overall, two RCTs reported pooled data under the category of “infection.^7,16^ ” Two studies provided separate data for urinary tract infections, intra-abdominal abscess, and wound infections.^17,18^ The CHIPOR trial reported abdominal wall abscesses and urinary tract infections as distinct adverse events.^14^ Similarly, the HORSE trial reported surgical site infections and urinary tract infections separately.^15^ As shown in Fig. 5, there were no significant differences in infections between the CRS group and the CRS followed by HIPEC group (OR 1.06; 95% CI 0.74–1.52; *p* = 0.74; *I*^2^ = 4%). Pneumonia and sepsis/septic shock were excluded from this analysis and were analyzed separately. The meta-analysis of five RCTs showed no significant differences in the incidence of sepsis/septic shock between the two groups (OR 1.37; 95% CI 0.54–4.49; *p* = 0.50; *I*^2^ = 0%).

A total of five RCTs reported data on hemorrhage.^7,14,16–18^ The meta-analysis showed no significant differences in the incidence of hemorrhage between the two groups (OR 1.56; 95% CI 0.62–3.87; *p* = 0.34; *I*^2^ = 0%).

Similarly, the incidence of lymphocele was similar in the group of patients who underwent CRS alone compared with the CRS followed by HIPEC group (OR 0.59; 95% CI 0.27–1.30; *p* = 0.19; *I*^2^ = 0%).

Finally, regarding gastrointestinal complications, four studies reported data on ileus/bowel (sub) occlusions. No statistically significant differences were found in any of the studies, nor with meta-analysis results for these adverse events between the HIPEC group and the non-HIPEC group (OR 1.56; 95% CI 0.62–3.87; *p* = 0.34; *I*^2^ = 0%) (Table 2).

### Risk of Bias

As summarized in Supplementary Material 4, overall, three trials in primary ovarian cancer indicated a low risk of bias,^14–16^ and one RCT raised concerns regarding the timing of randomization and the inclusion of patients changed during the accrual of the patients, as detailed in other sources.^7,9^ Finally, one trial raised some concerns regarding the selection of the reported results owing to deviations from the protocol.^18^

## Discussion

Our meta-analysis of RCTs demonstrated that the addition of HIPEC to surgery significantly improved PFS in the subgroup of patients with newly diagnosed advanced-stage EOC who received NACT. Conversely, we observed no significant difference in PFS between secondary CRS plus HIPEC and surgery alone in the setting of recurrent EOC where NACT had not been previously administered. Moreover, an overall risk of AKF of 10.6% was observed, with a significantly higher incidence of acute kidney injury compared with CRS alone. Regarding hematological alterations, a decrease in platelet count of any grade was significantly more frequent in the HIPEC group, but when focusing on grade 3 or worse events (platelet count < 50,000/mm^3^), no statistically significant difference was found. No significant increases were observed in general infections, sepsis/septic shock, hemorrhage, lymphocele, gastrointestinal complications, or liver dysfunctions between the HIPEC and non-HIPEC groups.

Compared with the most recent meta-analysis published in 2022 by Filis et al., which included only one RCT involving patients with recurrent EOC, our review incorporated two newly published RCTs that also enrolled relapsed patients.^14,15,21^ Among these two trials, the study by Fagotti et al. confirms the results of Zivanovic et al. in reporting no additional benefit in PFS treated with CRS plus HIPEC compared with CRS alone in patients with platinum-sensitive recurrent ovarian cancer who did not receive NACT. Conversely, the other study by Classe et al. included patients who underwent NACT prior to surgery and HIPEC, reporting a significant improvement in PFS in the HIPEC group.^14^ Therefore, the encouraging results of the CHIPOR trial on HIPEC may be applied to this select population.

In terms of toxicity and adverse events, the findings of this meta-analysis align with previous systematic reviews and meta-analyses that have compared CRS alone with CRS followed by HIPEC. Whether administered systemically or intraperitoneally, cisplatin is primarily excreted through the kidneys, which can result in its accumulation in the proximal renal tubules, leading to nephrotoxicity.^12,13^ Renal failure is a well-documented and frequent adverse effect associated with cisplatin treatment.^22^ In a meta-analysis of 12 RCTs, including 873 patients with intra-abdominal malignancies, compared with CRS alone, CRS plus HIPEC had greater nephrotoxicity (OR 0.45; 95% CI 0.21–0.98), while other adverse events did not differ significantly between the two groups.^23^ The frequency of renal failure in patients who underwent HIPEC varies across literature. In two studies it was reported to range between 40.4% and 48.4%.^12,13^ However, a recent meta-analysis by Santana et al., including a total of 4928 patients, reported a lower incidence of kidney injury of 8% following HIPEC.^11^ Similarly, Bakrin et al., in a multicenter retrospective cohort study of 566 patients, also reported an incidence of 8%.^24^ These findings align closely with our analysis, which showed an overall incidence of AKF of 10.6%. Moreover, the inclusion of sodium thiosulfate in the HIPEC administration protocol has been associated with a reduction in the frequency of kidney failure, confirming its role as an effective nephroprotective agent. In two RCTs analyzed in the present meta-analysis, sodium thiosulfate was incorporated during recruitment as part of a protocol amendment.^14,16^ This adjustment allowed for a direct comparison of outcomes before and after its implementation. Both trials reported a significant decrease in the incidence of renal adverse events in the HIPEC group following the introduction of sodium thiosulfate, highlighting its potential to mitigate nephrotoxicity in this context.^14,16^

Providing detailed information on PFS and the complications associated with HIPEC has significant practical implications, particularly in improving patient counseling when this treatment is offered in clinical trials, both in the upfront and recurrent settings, with and without NACT, where HIPEC remains an investigational approach. This understanding of adverse events also plays a critical role in weighing the risks and benefits of this approach when combined with survival outcome data. In our meta-analysis, we observed a significant benefit of HIPEC in terms of PFS in patients with EOC who had recently undergone systemic chemotherapy in the setting of primary advanced newly diagnosed disease. However, no statistically significant differences were observed with the addition of HIPEC to surgery in the setting of recurrent disease where NACT had not been previously administered. Therefore, considering the observed complication rates and the higher costs associated with HIPEC, the benefit of HIPEC might be limited to a selected population who have received NACT, both in first-line and second-line treatments, particularly in *BRCA* wild-type patients, as suggested by the post hoc analysis of the OVHIPEC-1 trial.^7^

### Strengths and Limitations

To the best of our knowledge, this is the first meta-analysis of RCTs that pools HR for PFS in patients undergoing CRS alone versus CRS followed by HIPEC in patients with relapsed EOC. In addition, unlike most meta-analyses in the literature, which generically pool adverse events of grade > 3 on the basis of CTCAE terminology, we sought to provide detailed data from high-quality RCTs on both surgery-related and chemotherapy-related complications. However, our study has several important limitations that require cautious interpretation of the results. First, the limited number of included RCTs resulted in fewer trials contributing data for any single complication, potentially reducing the statistical power of our analysis. Second, numerous confounding factors—such as variations in the use of sodium thiosulfate for nephroprotection, differences in cisplatin dosages, perfusion times and temperatures, and tumor characteristics—could influence the results. Third, the absence of data after primary debulking surgery limits the generalizability of our findings in the recurrent setting. These limitations underscore the need for additional high-quality studies in the future to provide more robust evidence for clinical practice.^25^

## Conclusions

In patients with newly diagnosed advanced-stage EOC treated with NACT, our results suggest that HIPEC combined with CRS appears to improve PFS compared with CRS alone. Conversely, no additional benefit in PFS was observed in the relapse setting in patients who did not receive NACT. Overall, CRS followed by HIPEC proved to be a safe procedure, with an adverse event profile comparable to that of CRS alone for most complications. However, closer monitoring is advised owing to the higher risk of acute kidney injury and reductions in platelet count.

## Supplementary Information

Below is the link to the electronic supplementary material.

Supplementary file1 (DOCX 1644 KB)
