# Supplementary material for: Cytoreductive Surgery Plus HIPEC in Recurrent or Newly Diagnosed Advanced Epithelial Ovarian Cancer: a Meta-analysis
Source: Ann Surg Oncol. 2025 Feb 4;32(5):3648–59. doi: 10.1245/s10434-025-16979-6 (PMC11976761; doi:10.1245/s10434-025-16979-6)

**Supplemental Material**

**Supplemental Material 1_ Search strategy**

PubMed

Results= 297 records

("hipecs"[All Fields] OR "hyperthermic intraperitoneal chemotherapy"[MeSH Terms] OR ("hyperthermic"[All Fields] AND "intraperitoneal"[All Fields] AND "chemotherapy"[All Fields]) OR "hyperthermic intraperitoneal chemotherapy"[All Fields] OR "hipec"[All Fields]) AND ("ovarian neoplasms"[MeSH Terms] OR ("ovarian"[All Fields] AND "neoplasms"[All Fields]) OR "ovarian neoplasms"[All Fields] OR ("ovarian"[All Fields] AND "cancer"[All Fields]) OR "ovarian cancer"[All Fields]) AND ("clinical trials as topic"[MeSH Terms] OR ("clinical"[All Fields] AND "trials"[All Fields] AND "topic"[All Fields]) OR "clinical trials as topic"[All Fields] OR "trial"[All Fields] OR "trial s"[All Fields] OR "trialed"[All Fields] OR "trialing"[All Fields] OR "trials"[All Fields])

Web of Science

Results= 390 records

((ALL=(HIPEC)) AND ALL=(ovarian cancer)) AND ALL=(trial)

EBSCOhost

Results= 47 records

(HIPEC AND "ovarian cancer" AND trial)

**Supplemental Material 2_Figure_ Hematological disorders (Grade >3)**

1. Anemia (Grade >3)

**
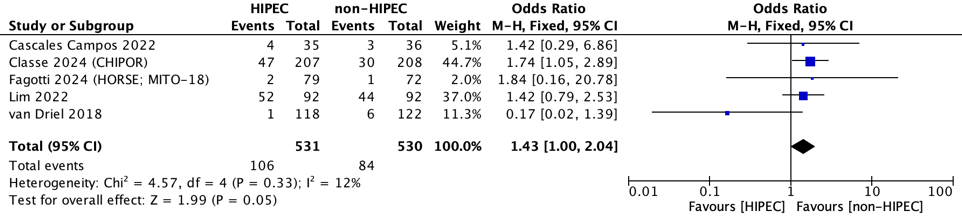
**

1. Platelet count decrease (Grade> 3)


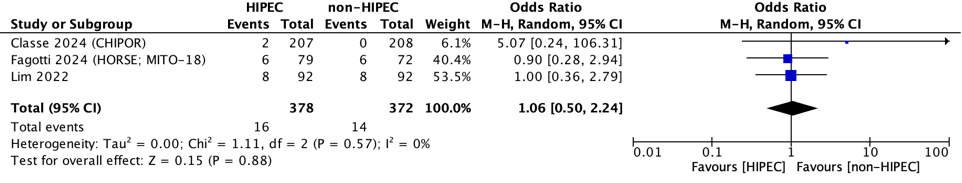


1. Neutrophil count decrease (Grade> 3)


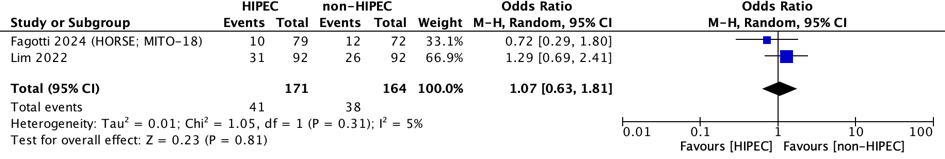


1. White blood cells decrease (Grade> 3)


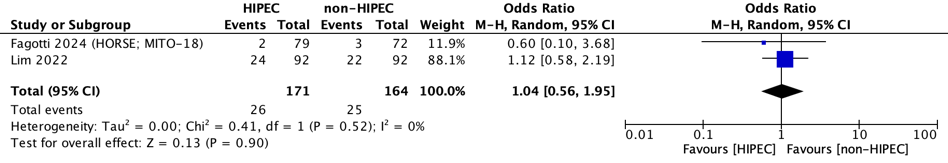


**Supplemental Material 3_Figure_ Other chemotherapy-related adverse events (Grade >3)**

1. Fatigue (Grade> 3)


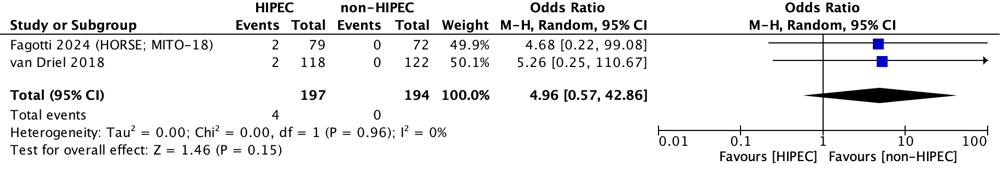


1. Nausea (Grade> 3)


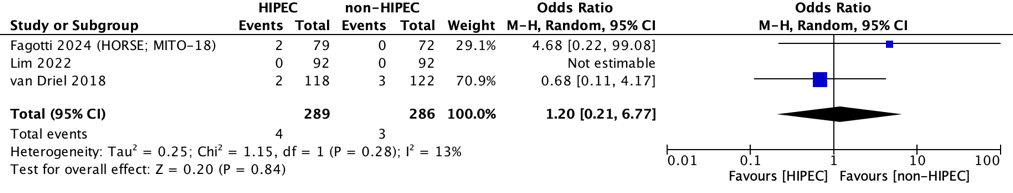


1. Diarrhea (Grade >3)


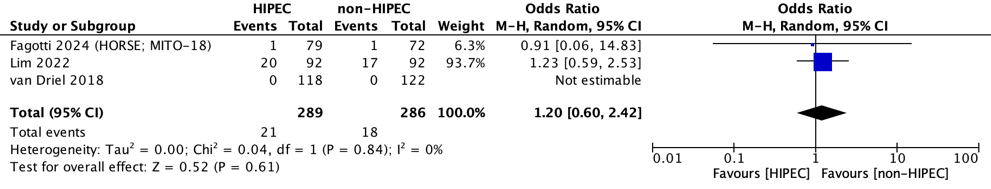


1. Vomiting (Grade >3)


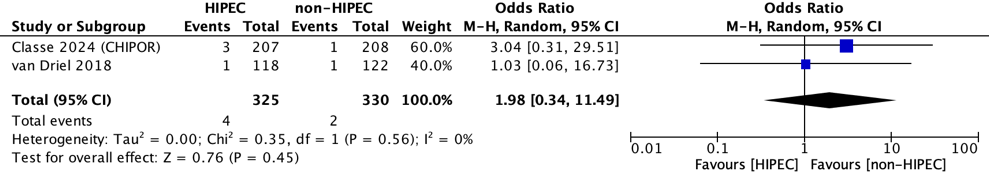


1. Constipation (Grade >3)


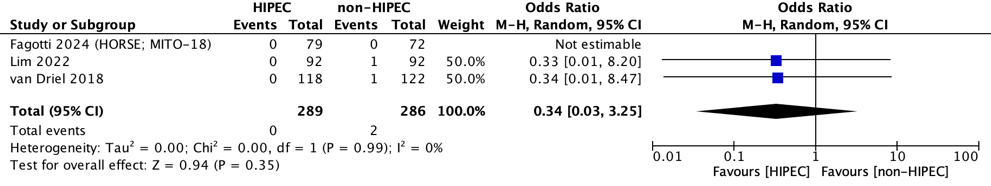


**Supplemental Material 4_ Risk of Bias Assessment**

Risk of bias graph: review authors' judgements about each risk of bias item presented as percentages across all included studies.


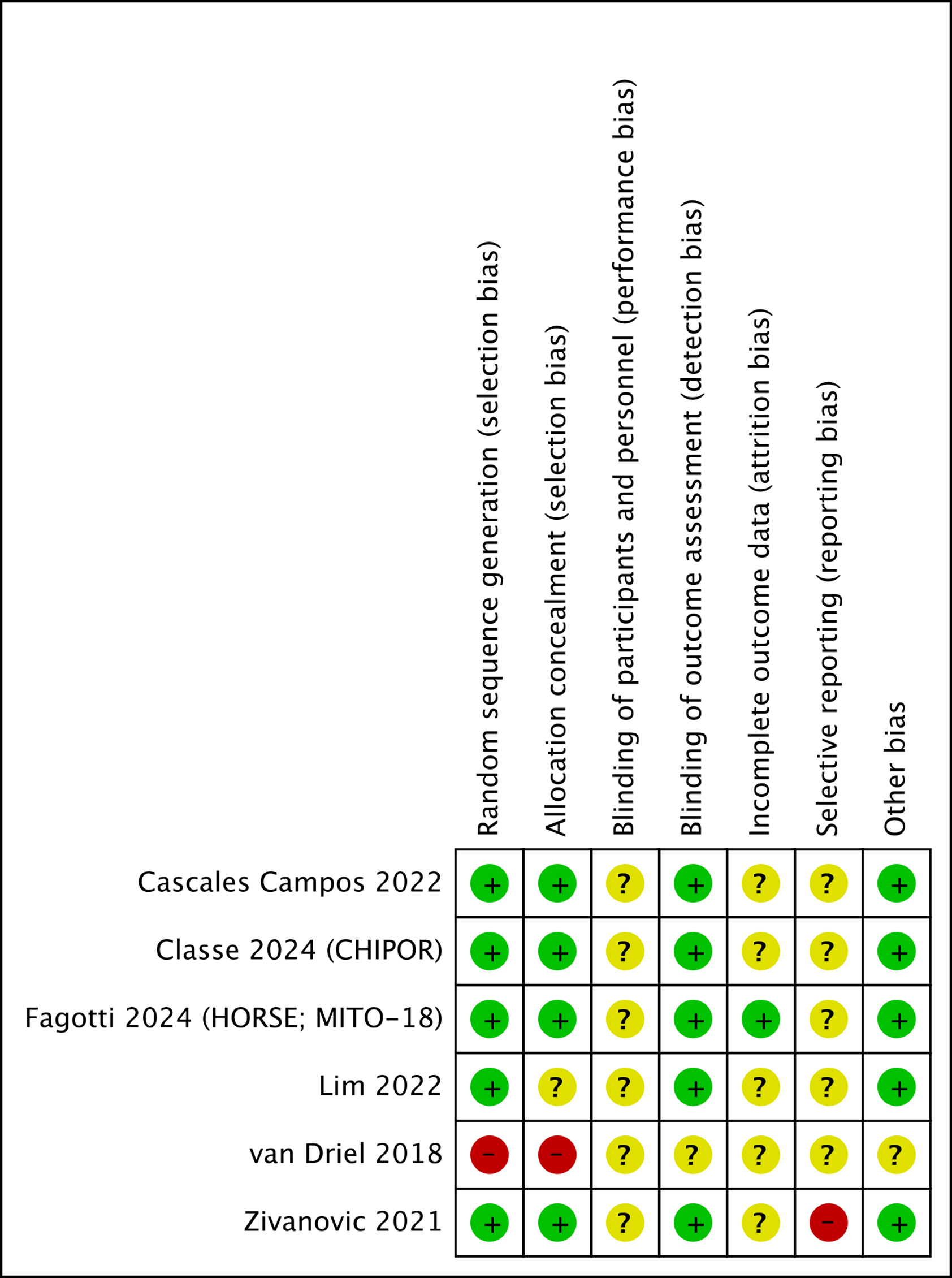

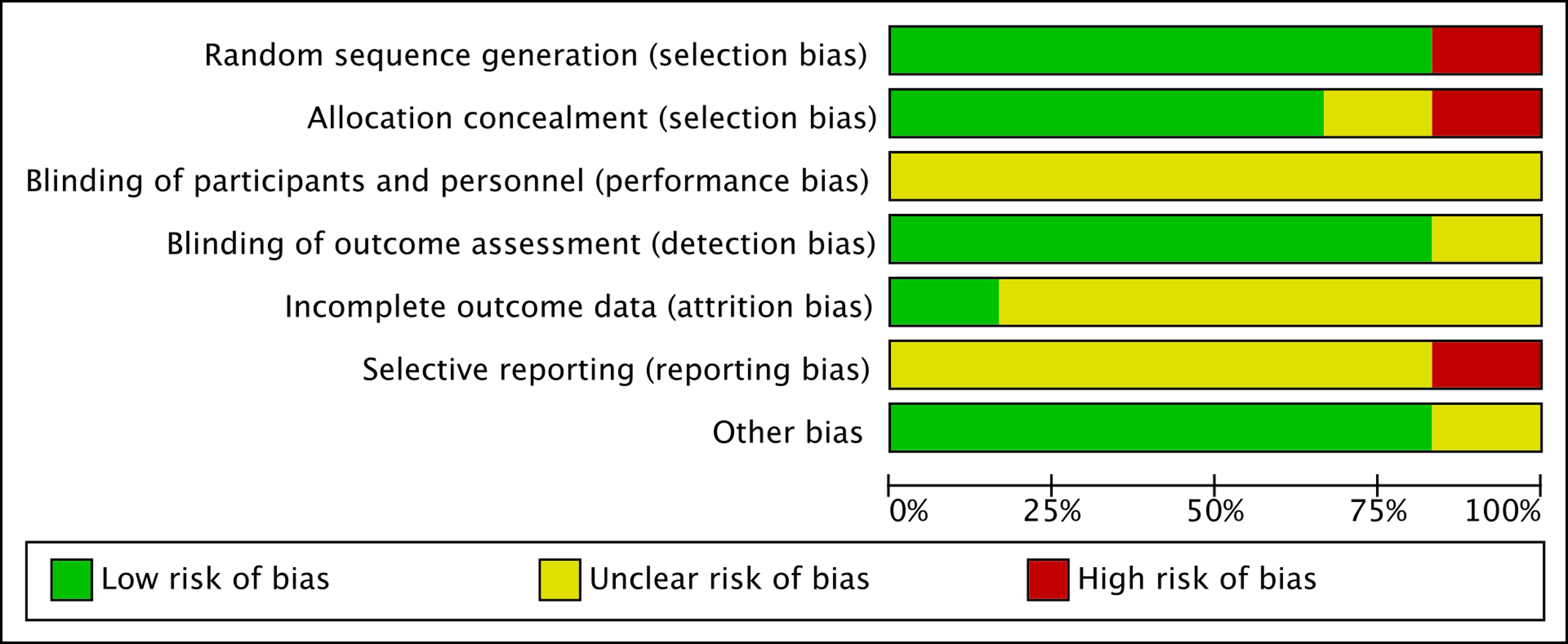

Supplement: Supplementary file 1 — Supplementary file1 (DOCX 1644 KB) [file 10434_2025_16979_MOESM1_ESM.docx]
